# You Are What You Read: The Belief Systems of Cyber-Bystanders on Social Networking Sites

**DOI:** 10.3389/fpsyg.2018.00365

**Published:** 2018-04-23

**Authors:** Angel N. M. Leung, Natalie Wong, JoAnn M. Farver

**Affiliations:** ^1^Department of Psychology and Centre for Psychosocial Health, The Education University of Hong Kong, Tai Po, Hong Kong; ^2^Department of Psychology, The Education University of Hong Kong, Tai Po, Hong Kong; ^3^Department of Psychology, University of Southern California, Los Angeles, CA, United States

**Keywords:** cyberbullying, cyber-bystanders, helping behavior, control beliefs, normative belief about helping, normative beliefs about aggression, social networking sites

## Abstract

The present study tested how exposure to two types of responses to a hypothetical simulated Facebook setting influenced cyber-bystanders’ perceived control and normative beliefs using a 4 cyberbully-victim group (pure cyberbullies, non-involved, pure cyberbullied victims, and cyberbullied-victims) × 2 condition (offend vs. defend) experimental design. 203 Hong Kong Chinese secondary school and university students (132 females, 71 males; 12 to 28; *M* = 16.70; *SD* = 3.03 years old) were randomly assigned into one of two conditions. Results showed that participants’ involvement in cyberbullying significantly related to their control beliefs about bully and victim assisting behaviors, while exposure to the two different conditions (offend vs. defend comments) was related to both their control and normative beliefs. In general, the defend condition promoted higher control beliefs to help the victims and promoted higher normative beliefs to help the victims. Regardless of their past involvement in cyberbullying and exposure to offend vs. defend conditions, both cyber-bullies and cyber-victims were more inclined to demonstrate normative beliefs to help victims than to assist bullies. These results have implications for examining environmental influences in predicting bystander behaviors in cyberbullying contexts, and for creating a positive environment to motivate adolescents to become “upstanders” in educational programs to combat cyberbullying.

## Introduction

The increase in cyberbullying on social network sites (SNS) has become a significant risk for the mental and physical health of adolescents in the United States and in many countries around the world (Patchin and Hinduja, 2012). Youth who are victims of cyberbullying suffer from numerous negative outcomes (anxiety, fear, depression, and low self-esteem) and they often struggle academically (Schneider et al., 2011). In a large-scale study of adolescents’ online behavior, Lenhart et al. (2011) found that while 88% had witnessed cyberbullying, most reported that they had ignored the bullying (90%), 67% had seen others join in, 21% had joined in themselves, and about 25% had defended the victim. Although prevalence rates have been well documented at present, we know little about the behavior of individuals who witness bullying in online contexts, and why or how they choose to respond.

Studies of conventional bullying in physical settings have demonstrated that bystanders can play an important role in accelerating or reducing the bullying (e.g., Salmivalli, 2010) by either reinforcing (O’Connell et al., 1999) or discouraging its occurrence (Lynn Hawkins et al., 2001). Cyberbullying differs from face-to-face bullying in the sheer number of people that can be involved due to the ease in which on-line posts, pictures, and videos can be stored, copied, and shared (e.g., Kowalski et al., 2014). Bystanders can only estimate how many others are witnessing the cyberbullying but they may not see how others react. These conditions can lead to a diffusion of responsibility, a characteristic of the bystander effect, which can result in the inhibition of supportive behavior (Latané and Darley, 1970; Thornberg, 2007). Moreover, online bystanders may not intend to reinforce cyberbullies’ behavior but they often inadvertently forward, comment on, or simply ‘like’ certain humiliating posts for fun (Macháčková et al., 2013). As a result, cyber-bystanders’ responses can contribute to the snowballing of cyberbullying by supporting cyberbullies’ goals to be dominant, admired, and powerful among their peers (e.g., Runions et al., 2013); and goals that motivate adolescents to perpetrate aggression (Salmivalli, 2010).

Throughout early and late adolescence, peers are powerful role models for the acquisition and maintenance of attitudes and beliefs about aggressive behaviors, and in particular bullying (Pepler et al., 2010; Salmivalli, 2010). A similar pattern of peer influence can also be expected to impact bystanders’ responses to cyberbullying. In fact, recent research has shown that cyber-bystanders will often defend cyberbullied victims or join in with the cyberbullying if other bystanders behave similarly (Bastiaensens et al., 2014, 2015). Guided by the Theory of Planned Behavior (TPB; Ajzen, 1991), we developed and carried out an experiment to investigate whether exposure to different types of other bystanders’ responses to cyberbullying (either helping the victim or assisting the bully) would alter individuals’ belief systems about how they would intervene in a cyberbullying situation.

Theory of Planned Behavior is a theoretical framework for predicting (and thus potentially changing) human social behavior. According to Ajzen (1991, p. 188), human behavior is determined by intention and encompasses three belief-based concepts: the attitude toward the behavior, subjective norms (i.e., perceived social pressure to perform or not perform the behavior), and perceived behavioral control over performing the behavior. In general, “the more favorable the attitude and subjective norm with respect to a behavior, and the greater the perceived behavioral control, the stronger should be an individual’s intention to perform the behavior.”

Theory of Planned Behavior has been applied to literally hundreds of past studies on health-related behavior (e.g., Godin and Kok, 1996), consumer choice (Taylor and Todd, 1995), college students’ alcohol use (Borsari and Carey, 2001), and more recently to cyberbullying. Most relevant here are the studies that examined the relations among subjective norms, behavioral control and cyberbullying behavior. Williams and Guerra (2007) found that adolescents’ cyberbullying behavior was related to their perception of peer approval of cyberbullying (i.e., their subjective norms). Similarly, in another two studies, Heirman and Walrave (2012) found that Flemish adolescents’ attitudes toward cyberbullying was the strongest predictor of their intention to engage in cyberbullying. DeSmet et al. (2016) also found that individuals’ ratings of self-efficacy to take action to stop cyberbullying predicted their intervening behavior. Based on the limited and preliminary evidence from cyberbullying research, it appears that individuals who have positive outcome expectancies which is presumably indicative of confidence in their ability to help, are more likely to intervene (DeSmet et al., 2016).

Social norms may also influence bystanders’ decision to join in with cyberbullying. A survey of Flemish youth conducted by Bastiaensens et al. (2016) found that those who thought their friends would approve of their cyberbullying felt more social pressure to join in when they witnessed instances of cyberbullying, and they were subsequently more likely to do so. This was not the case for classmates’ injunctive or moral norms who were sufficiently close to elicit social pressure. Cyberbullying-specific norms may influence multiple bystanders’ responses as mediated by the closeness of their peer group.

In the current study, we speculated that cyberbullying-specific norms and individuals’ confidence in their ability to intervene in cyberbullying would be shaped by the content they are exposed to on SNSs like *Facebook*. Most SNSs have built in algorithms to generate personalized content according to users’ tastes. For instance, *Facebook* shows posts or content that the users have agreed with or posts of friends with whom the users most interact. Bessi et al. (2016) found that on both *Facebook* and *YouTube* where internet users are exposed to different types of information, internet users tend to choose information that supports and adheres to their beliefs, and then they form polarized social groups who share similar views. Therefore, SNSs provide platforms that reinforce Echo Chamber Effects. Echo chamber effect was originally described as “the amplifying effects of opinion forming between politics, media, and the populace” (Key, 1966, cited in Pfeffer et al., 2014, p. 122). In the context of social media, it is now generally referring to the fact that SNS users tend to post and be connected with people sharing similar opinions, partly due to the built-in algorithms of SNS. In other words, the exposure to information on SNS would be likely to reinforce or reaffirm any prejudgement. In an integrative model predicting bystander behavior DeSmet et al. (2016) found that among a wide range of predictors, contextual factors such as class norms or media exposure seems to influence cyber-bystanders’ willingness to intervene. However, they did not account for the exposure to other bystanders’ behaviors to cyberbullying.

Accordingly, we argue that witnessing how others react to a cyberbullying incident on SNSs would create the Echo Chamber effect, which in turn would create a context that may change individuals’ perceived control and normative beliefs about either assisting the cyberbullies or helping the cyberbullied victims. For instance, individuals who are more inclined to help the victims may be more likely to have been exposed to groups or posts and opinions that reinforce such tendencies and vice versa; which may create a normative belief that it is acceptable to help victims or assist bullies. Similarly, perceived behavioral control or individuals’ perceived ease in intervening in cyberbullying may be shaped by observational learning.

Results from a recent meta-analysis (Kowalski et al., 2014) show that cyberbullying victimization is one of the strongest predictors of cyberbullying perpetration. In line with this finding, strong correlations between cyberbullying victimization and perpetration (*r* = 0.50 to 0.60) were reported in studies from both Western and Eastern cultures (e.g., Bauman et al., 2013; Wong et al., 2014). Furthermore, a recent attempt to divide a sample of 2186 adolescents into different cyberbullying involvement groups also suggested a strong co-occurrence between bullying victimization and perpetration in cyberspace (Mishna et al., 2012). These findings imply that retaliation or reactive bullying might explain some variance in cyberbullying behavior.

Currently, there are few studies investigating cyber-bystander behavior and virtually none have been conducted with a Hong Kong Chinese population. Hong Kong presents a unique context because at present, there is no statue law against cyberbullying in Hong Kong nor is it discussed or addressed in the curricula of most Hong Kong local schools or colleges. Moreover, given that Chinese traditional cultural values emphasize sensitivity to others and minimizing interpersonal conflicts, we can expect that Hong Kong Chinese adolescents will differ from their Western counterparts in how they notice or interpret cyberbullying, their attitudes toward it and how they respond (Li, 2008; Barlett et al., 2014). Since Hong Kong adolescents are not exposed to ways to respond to cyberbullying, their perceived control or confidence in their ability to help is likely to be lower than their Western counterparts. Cultural differences in endorsing a decision to intervene or not when witnessing cyberbullying, may also shape the subjective norms of Hong Kong adolescents. Finally, a recent study (Leung et al., in press) found that 58% of Hong Kong college students reported cyberbullying others; while 68% of those also reported being cyber-victimized themselves. These prevalence rates are comparable to those reported in Western settings. Therefore, it is important to carry out a study on cyber-bystanders’ behavior with Hong Kong adolescents.

The present study investigated how cyber-bystanders’ perceived control and normative beliefs may be influenced by exposure to two types of other bystanders’ responses in a hypothetical simulated *Facebook* setting using a 4 cyberbully-victim group (pure cyberbullies, non-involved, pure cyberbullied victims, and cyberbullied-victims) × 2 condition (offend vs. defend) experimental design. Participants were randomly assigned into one of two conditions and at the end of the experiment, their normative and control beliefs about bystander behaviors, and intention to assist bully/help victim, were measured and compared across two conditions and four groups of participants.

Based on the literature reviewed above, we hypothesized that:

(1) In the defend condition where other internet users support the victim, participants were expected to have higher (a) control and (b) normative beliefs to help the victim; whereas in the offend condition where other internet users support the bully or the bullying behavior, participants were expected to have higher (a) control and (b) normative beliefs to assist bully.
(2) In line with findings of both conventional bullying and cyberbullying (e.g., Heirman and Walrave, 2012; Burton et al., 2013), participants’ involvement in cyberbullying was expected to influence their control and normative beliefs about bystander behaviors. Therefore, regardless of the condition, cyberbullies were expected to demonstrate higher (a) control and (b) normative beliefs to support bullies (than their beliefs to help victims) whilst cyberbullied victims were expected to demonstrate higher (a) control and (b) normative beliefs to help victims (than their beliefs to support bullies).

## Materials and Methods

### Participants

The participants were 203 students (132 females, 71 males aged 12–28; *M* = 16.70; *SD* = 3.03 years old) who were recruited from a university and three secondary schools in Hong Kong. Prior to the data collection, participants provided informed consent and parental consent was obtained from those younger than 18 years old. At the end of the study, participants were de-briefed about the study’s purpose and rationale.

### Formation of Cyberbully-Victim Groups and Assignment to Experimental Condition

**FIGURE 1:**
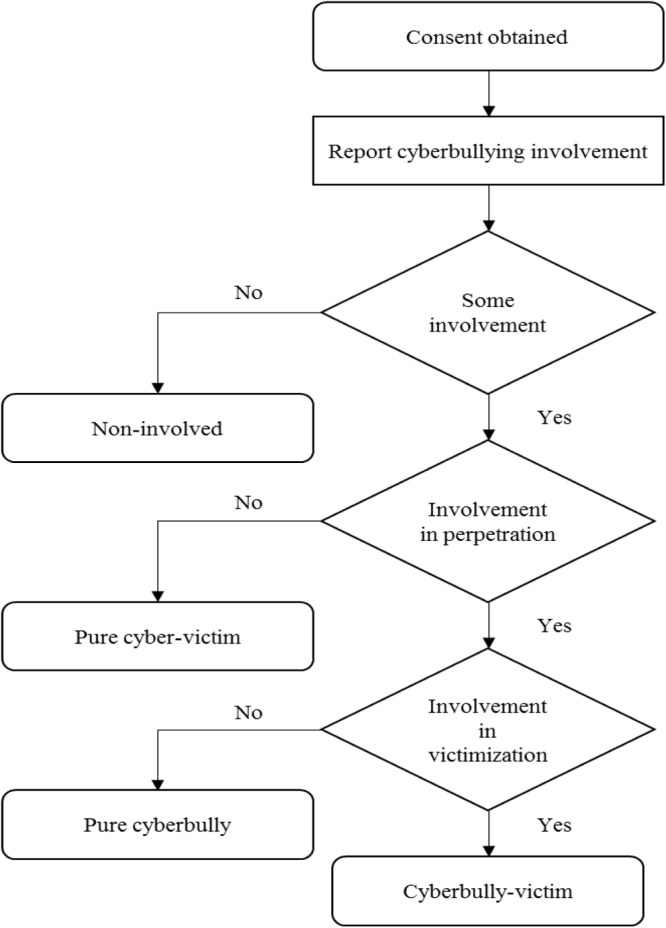
Cyberbully-victim group classification procedure.

Before the experiment began, participants completed a questionnaire about frequency of their experience with cyberbullying on various online platforms using a scale developed and tested in a prior study conducted in Hong Kong (Leung et al., in press), participants rated 10 items on a 5-point scale (1 = never; 5 = very frequently) to measure their cyberbullying behavior, (e.g., *I gossip or say mean things about others on the internet;* alpha = 0.91), and their cyberbullying victimization, (e.g., *Others gossip or say mean things about me on the internet*; alpha = 0.91). Based on their responses, participants were classified into one of four cyberbully-victim groups: non-involved, pure bullies, pure victims, and cyberbully-victims using mean score as cut-off points for the two scales (see **Figure 1**).

Participants with a mean score of 1 (i.e., score of 1 = no experience as a bully or a victim) for both the cyberbullying perpetration and victimization scales were classified as *non-involved*; a mean score of 1 for cyberbullying victimization but more than 1 for cyberbullying perpetration were classified as *pure cyberbullies*; a mean score of 1 for cyberbullying perpetration but more than 1 for cyberbullying victimization were classified as *pure cyberbullied victims*; and a mean score of more than 1 for both cyberbullying perpetration and victimization were classified as *cyberbully-victims*. After classifying participants into the four cyberbully-victim groups, about half of each group were randomly assigned to either a *defend* or an *offend* experimental condition.

### Experimental Procedure

Participants attended to a hypothetical *Facebook* bullying scenario on their computers in the computer room in their schools or at the university. Both experimental conditions began with a simulated *Facebook* webpage where a person posted a demeaning picture of a teenager named Alex Wong for his friends to see. Alex Wong then left a comment to ask the perpetrator to please take away the post.

Participants in the *offend* condition saw comments that supported the bullying behavior (e.g., *Serves you right Alex*!) or further offended the victim (e.g., *Alex has always been a loser anyway*); participants in the *defend* condition saw comments against bullying behavior (e.g., *Can the person who posted this picture please take down the post*?) or that supported the victim (e.g., *Poor Alex. Have you thought of his feelings*?). The *Facebook* page auto-refreshed every few minutes, and a notification icon appeared at the top right hand corner of the page each time a new comment was added. Participants were asked to read each comment carefully. Approximately, 200 comments were displayed in both conditions and the whole experiment took about 20 min. After viewing the comments relevant to their experimental condition, participants completed several questionnaires to measure their normative and control beliefs about their intention to help victims, and to assist the bullies.

### Measures for Normative and Control Beliefs About Bystanders’ Behaviors

We developed a questionnaire based on previous research on cyberbullying and peer relations among Hong Kong adolescents of varied ages to measure normative and control beliefs about bystanders’ behaviors. The items were adapted and operationalized as recommended by Fishbein and Ajzen (2011).

Normative beliefs about bystander behaviors consisted of two 5-item parallel scales about bully assisting (e.g., *I think people who matter to me would appreciate it if I assist the cyberbully*; alpha = 0.82) and victim helping (e.g., *I think people who matter to me would appreciate it if I help the cyberbullied victim*; alpha = 0.82) in a cyberbullying situation.

Control beliefs about bystander behaviors consisted of two 7-item parallel scales about bully assisting (e.g., *I think cyberbullying others is easy*; alpha = 0.77) and victim helping in a cyberbullying situation (e.g., *I think helping a cyberbullied victim is easy*; alpha = 0.81).

## Results

### Cyberbullying Involvement Across School Levels and Gender

As shown in **Table 1**, 45% of the participants had cyberbullied others at least once prior the administration of the questionnaire, while 56% had been cyberbullied. This rate of involvement in cyberbullying is similar to rates reported in Western settings. For instance, after reviewing 73 studies on cyberbullying, Patchin (2013) found that the prevalence rates across all the studies ranged from 2.3 to 72% for cyber-victimization; and from 1.2 to 44.1% for perpetrating cyberbullying. Results of the current study show that cyberbullying prevalence rates for Hong Kong students is comparable. To examine the differences across cyberbully-victim groups, we have divided our participants into 4 groups basing on the procedure outlined above. **Table 2** displays the means and standard deviations of cyberbullying involvement by cyberbully-victim group.

**Table 1:** Means and standard deviations for reports of cyberbullying involvement by school level and gender.

	Whole	Secondary	College	Male	Female
	(*N* = 203) *M* ±*SD*	(*N* = 110) *M* ±*SD*	(*N* = 93) *M* ±*SD*	(*N* = 71) *M* ±*SD*	(*N* = 132) *M* ±*SD*
Perpetration	1.24 ± 0.442	1.21 ± 0.444	1.28 ± 0.441	1.36 ± 0.595	1.18 ± 0.316
Victimization	1.45 ± 0.595	1.52 ± 0.637	1.37 ± 0.533	1.54 ± 0.655	1.40 ± 0.557

**Table 2:** Means and standard deviations of cyberbullying involvement by cyberbully-victim group.

	Whole sample	Non-involved	Pure cyberbullies	Pure cybervictims	Cyberbully-victims
	(*N* = 203) *M* ±*SD*	(*N* = 65) *M* ±*SD*	(*N* = 25) *M* ±*SD*	(*N* = 49) *M* ±*SD*	(*N* = 64) *M* ±*SD*
Perpetration	1.24 ± 0.442	1 ± 0	1.22 ± 0.126	1 ± 0	1.68 ± 0.569
Victimization	1.45 ± 0.595	1 ± 0	1 ± 0	1.55 ± 0.366	2.01 ± 0.648

A 2 (school level: secondary or college; between-subject factor) × 2 (gender: male or female; between-subject factor) × 2 (type of cyberbullying involvement: cyberbullying victimization or perpetration; within-subject factor) repeated measures ANOVA was conducted to examine the effects of school level and gender on cyberbullying involvement.

The results revealed a significant main effect of cyberbullying involvement, Pillai’s trace *F*(1,199) = 33.323, *p* < 0.001, $\eta_{p}^{2}$ = 0.143; and a significant interaction between school level and type of cyberbullying involvement, Pillai’s trace *F*(1,199) = 8.238, *p* = 0.005, $\eta_{p}^{2}$ = 0.040. The interaction between gender and cyberbullying involvement was non-significant. To examine the 2-way interactions between school level and cyberbullying involvement, 2 pairs of *t*-tests were performed. The results indicated that the school level did not have a significant independent effect on cyberbullying perpetration or victimization.

To examine hypothesis 1(a) and 2(a), a 2 (condition: defend or offend; between-subject factor) × 4 (cyberbully-victim group: non-involved, pure cyberbullies, pure cyberbullied victims, or cyberbully-victims; between-subject factor) × 2 (type of control belief: belief about assisting the bully or belief about helping the victim; within-subject factor) repeated measures ANOVA was conducted to examine the effect of the defend/offend conditions and cyberbully-victim groups on participants’ control beliefs about bystander behaviors on control beliefs. The results revealed a significant 2-way interaction between cyberbully-victim group and type of control beliefs, Pillai’s trace *F*(3,195) = 4.295, *p* = 0.006, $\eta_{p}^{2}$ = 0.062; and a significant 2-way interaction between defend/offend condition and type of control belief, Pillai’s trace *F*(1,195) = 6.996, *p* = 0.009, $\eta_{p}^{2}$ = 0.035.

**FIGURE 2:**
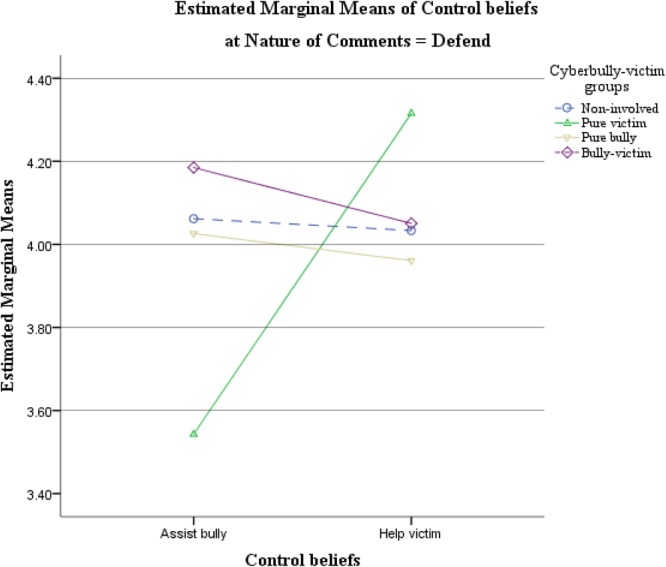
Estimated marginal means of the 2-way interaction between cyberbully-victim group and type of control beliefs about bystander behaviors in the defend condition.

**FIGURE 3:**
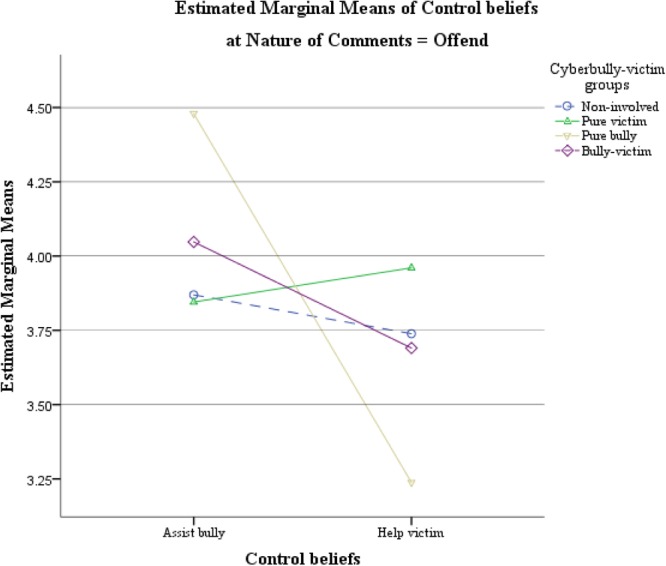
Estimated marginal means of the 2-way interaction between cyberbully-victim group and type of control beliefs about bystander behaviors in the offend condition.

To examine the 2-way interaction between defend/offend conditions and control beliefs, two pairs of *post hoc t*-tests were performed. Our results indicated that participants generally reported a significantly higher level of control beliefs about victim helping in defend than in offend condition, *t*(201) = 2.986, *p* = 0.003. Control beliefs about assisting the bullies, on the other hand, were similar for the two conditions, *t*(201) = -0.179, *p* = 0.858. Therefore, hypothesis 1(a) was partly supported. The defend condition promoted higher control beliefs to help the victims but participants did not have higher control beliefs to assist the bullies in the offend condition. **Figures 2, 3** illustrate the estimated marginal means of the 2-way interaction between cyberbully-victim group and control beliefs about bystander behaviors in defend and offend conditions separately.

**FIGURE 4:**
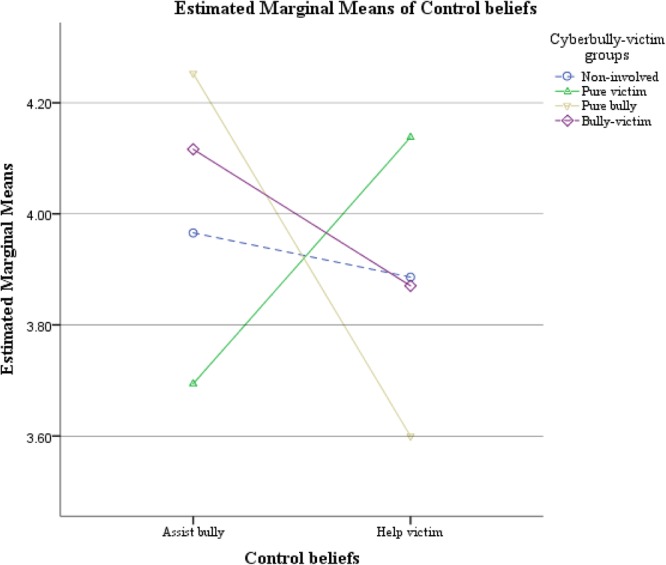
Estimated marginal means of the 2-way interaction between cyberbully-victim group and type of control beliefs about bystander behaviors.

To examine the 2-way interaction between cyberbully-victim groups and control beliefs about bystander behaviors, four pairs of *post hoc t*-tests were performed. The results indicated that pure cyberbullied victims reported higher control beliefs about victim helping than that of bully assisting, *t*(48) = 2.367, *p* = 0.022. On the other hand, pure cyberbullies generally reported high control beliefs about bully assisting than for victim helping, *t*(24) = 2.131, *p* = 0.044. The difference between control beliefs about assisting the bully and helping the victim were not significant among participants who were classified as non-involved, *t*(64) = 0.589, *p* = 0.558 and as cyberbully-victims, *t*(63) = 1.335, *p* = 0.187. Bonferroni correction was not adopted here as we have preplanned hypotheses, while Bonferroni adjustments may boost type II error (Armstrong, 2014). **Figure 4** illustrates the estimated marginal means of the 2-way interaction between cyberbully-victim group and type of control beliefs. Therefore, regardless of the nature of the condition (i.e., being exposed to offend or defend conditions), cyberbullies demonstrated higher control beliefs about supporting bullies; whilst cyberbullied victims demonstrated higher control beliefs about helping victims. Hypothesis 2(a) was supported.

**FIGURE 5:**
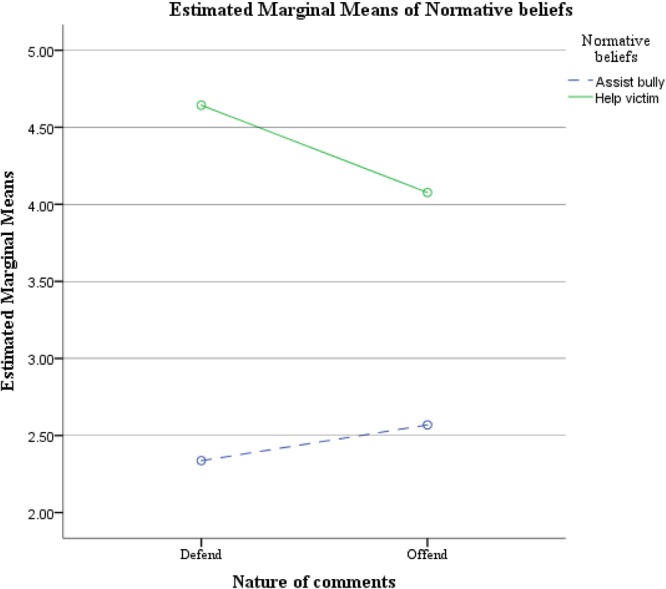
Estimated marginal means of the 2-way interaction between defend/offend condition and type of normative beliefs about bystander behaviors.

To examine hypotheses 1(b) and 2(b), a 2 (condition: defend or offend; between-subject factor) × 4 (cyberbully-victim group: non-involved, pure cyberbullies, pure cyberbullied victims, or cyberbully-victims; between-subject factor) × 2 (type of normative belief: belief about assisting the bully or belief about helping the victim; within-subject factor) repeated measures ANOVA was conducted. The interaction between cyberbully-victim group and type of normative belief was non-significant, indicating that cyberbullies and cyberbullied-victims did not demonstrate significant differences in the two types of normative beliefs. Therefore, hypothesis 2(b) was rejected. However, the results did reveal significant main effect for the two types of normative beliefs (i.e., assisting the bully or helping the victim), Pillai’s trace *F*(1,195) = 220.667, *p* < 0.001, $\eta_{p}^{2}$ = 0.531, and a significant interaction between defend/offend condition and type of normative belief, Pillai’s trace *F*(1,195) = 9.661, *p* = 0.002, $\eta_{p}^{2}$ = 0.047. **Figure 5** illustrates the estimated marginal means of the 2-way interaction between defend/offend condition and type of normative belief.

To examine the 2-way interaction between defend/offend conditions and normative beliefs about bystander behaviors, and the main effect of the two types of normative beliefs, three pairs of *t*-tests were performed. In terms of normative beliefs about bystander behaviors across conditions, participants generally reported a significantly higher level of normative beliefs about helping the victims in defend than in offend condition, *t*(201) = 3.362, *p* = 0.001. Normative beliefs about assisting the bully, on the other hand, were similar between the two conditions, *t*(201) = -1.143, *p* = 0.255. Therefore, hypothesis 1(b) was only partly supported. The defend condition promoted higher normative beliefs to help the victims, but the offend condition did not promote higher normative beliefs to assist the bullies. Finally, the results indicated that participants generally reported a higher level of normative beliefs about helping victims than that of assisting the bully, *t*(202) = 15.313, *p* < 0.001. To conclude, regardless of conditions, and across four cyberbully-victim groups, participants were more inclined to have a higher level of normative beliefs about victim helping.

## Discussion

The present study investigated how bystanders’ responses to cyberbullying might differ based on how others react to bullying scenarios in a simulated experiment with a 4 cyberbully-victim groups × 2 experimental conditions. The results showed that our sample could be effectively divided into four distinct cyberbully-victim groups, which demonstrated different bystander behavioral tendencies. In line with studies of both conventional bullying and cyberbullying (e.g., Heirman and Walrave, 2012; Burton et al., 2013), participants’ involvement in cyberbullying was generally found to be significantly related to their control beliefs about bully and victim assisting behaviors, while exposure to two different conditions (offend vs. defend comments) was significantly related to both their control and normative beliefs. Regardless of their past involvement in cyberbullying and exposure to offend vs. defend conditions, both cyber-bullies and cyber-victims were more inclined to demonstrate normative beliefs to help victims than to assist bullies.

The interaction of individual and environmental factors was examined using the classification of cyberbully-victim groups and experimental manipulation. Consistent with our hypotheses, we found a significant interaction between the experimental conditions and their perceived control beliefs about bystander behaviors. This finding demonstrates the significance of environmental influences, specifically exposure to other people’s comments, in predicting bystanders’ interventions. These results have implications for the importance of examining environmental influences to predict bystander behaviors in cyberbullying.

Clearly, bystanders’ behaviors can contribute or alter the dynamic of a cyberbullying scenario by motivating other spectators to help the victim. According to TPB, individuals’ control and normative beliefs predict individuals’ intentions and in turn predict their future behavior. This finding supports the role of environmental factors in the design and evaluation of educational programs for cyberbullying. Future anti-cyberbullying programs designed to help victims of cyberbullying, should emphasize how other bystanders can show support for the victims by leaving positive and supporting comments on SNSs, which can create a positive environment to motivate adolescents to become “upstanders” instead of passively witnessing cyberbullying. Furthermore, it is possible that the defend condition seemed to be more effective in shaping both control and normative beliefs about helping victims than in the offend condition, is that helping victims is a more socially desirable behavior. Nevertheless, it was alarming to find that cyberbullies were more inclined to have higher control beliefs to assist bullies when exposed to the offend condition. In other words, adolescents who have high rates of prior involvement in cyberbullying may be more prone to be further reinforced in their control beliefs to assist bullies than to help the victims.

Nowadays, adolescents are easily exposed to Echo Chamber Effects when they access any kinds of SNSs. Individuals’ opinions are more likely be further reinforced because of the built in functions of several SNSs that support the beliefs of users based on big data analysis. This current study was a short, yet effective replication of the everyday encounters of internet users, and it shows that a short period of exposure to an artificial experimental condition can result in a significant change in adolescents’ control beliefs. This also points to the importance of carefully selecting and accessing information and opinions from SNSs and provides insight about how educators can help the next generation to critically evaluate the opinions they access from SNSs, and to carefully reflect on whether highly polarized opinions may affect individuals’ control beliefs about cyberbullying behavior.

The results also show that normative beliefs about bystander behavior was a relatively stable belief construct as compared to control beliefs. Therefore, only part of hypothesis 1(b) was supported while hypothesis 2(b) was rejected. Only the defend condition promoted higher normative beliefs to help the victims. However, regardless of condition and the four cyberbully-victim groups, participants were more inclined to have higher levels of normative beliefs about victim helping. This finding may be attributed to the fact that we measured normative beliefs based on how participants thought people who matter to them would appreciate their efforts to assist victims or join the bullies. Bully-helping behavior is by no means socially desirable, especially from the perspective of “people who matter” to the participants as these can be their parents, peers, or teachers. This applies to all participants regardless of their past involvement in cyberbullying. Therefore, it is not surprising that participants were only sensitive in the defend condition to help victims, but not in the offend condition to assist bullies. In designing future educational programs, we believe that including perspectives of other important and significant people in adolescents’ lives could make a difference in their responses and behaviors. If adolescents believe that people who matter to them, especially their peers, find defending and helping victims as a socially acceptable behavior, they are highly likely to adhere to this normative belief, which in turn, may increase their future positive bystander behaviors. Additional research is needed to support such a speculation.

The results on gender differences and cyberbullying are inconclusive. While some studies have suggested that cyberbullying perpetration is more prevalent among boys (Li, 2006; Dehue et al., 2008) and that girls are more often victims (Smith et al., 2008), other studies have found no gender differences (e.g., Ybarra and Mitchell, 2004; Williams and Guerra, 2007; Hinduja and Patchin, 2008; Slonje and Smith, 2008). Similarly, in the present study, we found no gender differences in cyberbullying involvement, a finding that needs further replication.

The present study is not without limitations. First, we only evaluated the effect of bystanders’ responses to cyberbullying on a single dimension, i.e., to help the victim or to assist the bully. Therefore, further differentiation of bystander behaviors is needed to draw conclusions on how bystanders’ interventions in cyberbullying. Second, *Facebook* was chosen as the platform for the hypothetical cyberbullying scenario because of its popularity among youth in Hong Kong. However, these results may not generalize to other SNSs as functionality and habits might be slightly different across platforms.

Despite these limitations, this study has made important contributions to our understanding of cyberbullying. Practically, we have refined the integrative model of behavioral prediction and developed a useful tool to help us examine bystander behaviors in cyberbullying using simulated scenarios. This tool can be used in a non-intrusive experiment by other researchers and educators in the future. Theoretically, the study has demonstrated the importance of accounting for the interaction of peer influence and individual factors in predicting bystander responses to cyberbullying. Background influences, such as exposure to other people’s comments seemed to influence participants’ inclination to help the victim or the bully. This result echoes and addresses the concerns raised in the literature (e.g., DeSmet et al., 2016), namely that exposure to the mass media comments could be an important predictor in explaining bystander behavior without providing empirical evidence. In the future, besides understanding what cause cyber-bullies to perpetrate cyberbullying, and increasing the resilience of cyber-victims, it is important to include to include the third element in future educational regimes. “You are what you read,” reading defending comments for victims on SNS promotes students’ positive beliefs toward helping cyber-victims. Such findings have important implications for cyberbullying research and future education efforts.

## Ethics Statement

Ethics approval was obtained from the Human Research Ethics Committee of The Education University of Hong Kong. Informed consent were obtained before the start of the study.

## Author Contributions

AL conceived and designed the study, collected and analyzed the data. NW analyzed the data. AL, NW, and JF wrote the paper.

## Conflict of Interest Statement

The authors declare that the research was conducted in the absence of any commercial or financial relationships that could be construed as a potential conflict of interest.
